# TROP-2 Promotes Cell Proliferation via the AKT-Mediated PKC**α** Pathway and Is a Novel Target for Antibody-Drug Conjugates in Penile Carcinoma

**DOI:** 10.32604/or.2025.066184

**Published:** 2025-11-27

**Authors:** Yi Tang, Minyi Situ, Zhiming Wu, Yanjun Wang, Xinpei Deng, Zhicheng Liu, Shengjie Guo, Qianghua Zhou, Gangjun Yuan, Xingliang Tan, Kai Yao

**Affiliations:** 1 Department of Urology, Sun Yat-sen University Cancer Center, Guangzhou, 510060, China; 2 State Key Laboratory of Oncology in Southern China, Guangzhou, 510060, China; 3 Collaborative Innovation Center of Cancer Medicine, Guangzhou, 510060, China; 4 State Key Laboratory of Oncology in South China, Guangdong Provincial Clinical Research Center for Cancer, Guangzhou, 510060, China; 5 Department of Urology Oncological Surgery, Chongqing University Cancer Hospital, Chongqing, 400030, China

**Keywords:** Penile squamous cell carcinoma, trophoblast cell-surface antigen-2, protein kinase B, cell proliferation, targeted therapy

## Abstract

**Background:**

Current chemotherapy treatments, including the TIP (Taxol, Ifosfamide, Cisplatin) regimen, have shown limited effects but strong side effects in advanced Penile squamous cell carcinoma (PSCC) patients. Trophoblast cell-surface antigen-2 (TROP-2) is a novel target for antibody-drug conjugate (ADC) drugs and has been proven to be effective in several human cancers. This study aimed to explore the biological function and potential of the ADC target in PSCC cells.

**Methods:**

A total of 196 PSCC tumor tissue specimens and clinicopathological data were collected. TROP-2 expression was detected by IHC, and the correlation between TROP-2 expression and the clinicopathological data of patients was analysed through statistical methods. Then, a series of *in vivo* and *in vitro* experiments were conducted to investigate the mechanism by which TROP-2 promotes PSCC cell proliferation. Additionally, the efficacy of TROP-2-targeted ADC and cisplatin was tested in PSCC cell lines and animal models.

**Results:**

Immunohistochemistry (IHC) revealed that TROP-2 was highly expressed in 60.2% of tumor specimens, and statistical analysis revealed that high TROP-2 expression correlated with advanced pT and pN stages and extranodal extension (ENE), as well as poor prognosis. Knockdown of TROP-2 inhibited the proliferation of PSCC cells both *in vivo* and *in vitro*, and this inhibitory effect was later proven to be mediated by protein kinase B (AKT), which is involved in the protein kinase C alpha (PKCα) signalling pathway. Furthermore, compared with cisplatin, TROP-2 targeted ADC could achieve an equivalent inhibitory effect on the proliferation of PSCC both *in vivo* and *in vitro*.

**Conclusion:**

TROP-2 promoted PSCC cell proliferation via AKT/PKCα-dependent pathway, and TROP-2-targeted ADC-drug has a gratifying inhibitory effect on PSCC proliferation both *in vivo* and *in vitro*.

## 1 Introduction

Penile squamous cell carcinoma (PSCC) is a severe urogenital malignancy. Patients with advanced PSCC, particularly those with pelvic lymph or organ metastasis, generally have a poor prognosis and derive limited benefit from surgery alone [1,2].

Adjuvant chemotherapy combined with radical inguinal lymph node dissection is the first-line therapy for advanced PSCC patients [2–4]. However, traditional cisplatin-based chemotherapies such as TIP (Taxol, Ifosfamide, Cisplatin) have a moderate objective response (OR) of 40% and they also bring serious side effects, including neutropenia, anaemia and thrombocytopenia, with 40% of patients showing grade 3 or greater toxicity [5,6]. Thus, novel treatments for PSCC are urgently needed.

Antibody-drug conjugates (ADCs), such as human epidermal growth factor receptor 2 (HER-2), nectin cell adhesion molecule 4 (Nectin-4), and epidermal growth factor receptor (EGFR)-targeted ADC drugs, have been explored recently and have been shown to be effective in several human cancers [7–9]. TROP-2, a transmembrane calcium signal transducer engaged in multiple cell signaling pathways, has recently emerged as a novel molecular target for ADC drugs [10–12]. Notably, sacituzumab govitecan (SG), which is a TROP-2 ADC drug that has been investigated in various clinical trials, has been validated to be an effective agent for urothelial carcinoma and epithelial carcinoma [13–15]. But the function of TROP-2 in PSCC remains uncertain and requires further exploration.

This study aims to reveal the biological function and prognostic value of TROP-2 in PSCC and its potential in ADC therapy for PSCC patients. In this study, we found that high TROP-2 expression is associated with poor prognosis and adverse clinicopathological features. Furthermore, we evaluated the impact of TROP-2 ADC on PSCC cell proliferation, finding that it effectively inhibited proliferation in both *in vitro* and *in vivo*.

## 2 Methods

### 2.1 Patient Cohort, Tissue Sample, and Research Ethics

Our study analyzed 198 patients diagnosed with PSCC from 2000 to 2022, and Table S1 lists the characteristics of patients included in this study. Clinical, pathological, and survival data for each patient were extracted based on the 8th edition (2017) of the TNM Staging System for Penile Cancer [16]. The Sun Yat-sen University Cancer Center (SYSUCC) Ethics Committee (SZR2023-062) approved this study, and informed consent was obtained.

### 2.2 Immunohistochemistry (IHC) Assay

The expression of TROP-2 was determined by immunohistochemistry using a purified polyclonal antibody (CST, E8Y8S, 1:1000, Danvers, MA, USA). Initially, tissue sections embedded in paraffin from 198 PSCC patients underwent deparaffinization in xylene, followed by rehydration using an alcohol gradient of 100%, 90%, 80%, 70%, 50% and 0%. We performed antigen retrieval using 10 mM pH 6.0 citrate buffer for 15 min, followed by a 15-min blocking of nonspecific antigens with QuickBlock™ Blocking Buffer (Beyotime, Shanghai, China). To prevent nonspecific binding, sections of patients were pre-treated with 10% normal goat serum (Beyotime, Shanghai, China), and it was incubated with TROP-2 antibody for a night. Then it was incubated with HRP-labeled goat anti-rabbit secondary antibody (Beyotime, A0208, 1:1000) for 2 h. IHC staining was subsequently visualized using a peroxidase EnVision Detection Kit (Dako, Glostrup, Denmark, K5007). Two independent pathologists, blinded to patient information, assessed the immunostaining. We assigned scores based on positive cell percentage: 0% is 0, 1%–33% is 1, 34%–66% is 2, and 67%–100% is 3. The staining intensity for TROP2 staining was scored on a scale from 0 to 3, where no staining was scored with 0, weak staining was scored with 1, moderate staining was scored with 2, and strong staining was scored with 3. Final scores were determined by multiplying the score of positive cells by their intensity. X-Tile software (version 3.6.1) was used to determine the cut-off value. According to the results (Fig. S1), the cut-off value for the TROP-2 IHC score was 4 points, and TROP-2 expression was divided into low (0–4) or high (5–9) according to the score.

### 2.3 Cell Lines, Culture Conditions, and Transfection Methods

The PSCC cell lines Penl1, Penl2, and 149RM were developed in our laboratory, as detailed in an earlier study [17]. The human epidermis keratinocyte (HaCaT), 149RCA, and LM156 cell lines were obtained from the Type Culture Collection of the Chinese Academy of Sciences (Shanghai, China). These cell lines have passed the Short Tandem Repeat (STR) authentication and were not contaminated by mycoplasma. This cell line was cultured in DMEM supplemented with 10% FBS (Gibco, Waltham, MA, USA). Penl2 and 149RM cell lines were transfected with negative control (NC) shRNA plasmids, shTROP-2 interference plasmids, TROP-2 overexpression plasmids, and negative vector plasmids. The following sequences are effective sequences of plasmids: TROP-2-sh1, CCGGGCTCATCTATTACCTGGACGACTCGAGTCGTCCAGGTAATAGATGAGCTTTTTT; TROP-2-sh2, CCGGCGTGGACAACGATGGCCTCTACTCGAGTAGAGGCCATCGTTGTCCACGTTTTTT.

### 2.4 Western Blot (WB)

Proteins of PSCC cells and patients’ tissue were extracted to examine the transfection efficiency of plasmids and the changes of relevant protein using RIPA lysis buffer (Beyotime), followed by separation via 10% SDS-PAGE (EpiZyme, Cambridge, MA, USA), and each lane was loaded with 10 μL protein. Extracted proteins were transferred to PVDF membranes (Pierce Biotechnology, Waltham, MA, USA), and 5% milk was used to prevent nonspecific binding for 1 h. Then, PVDF membranes were incubated with primary antibodies at 4°C overnight, and details of these antibodies are listed in Table S2. Subsequently, the membranes underwent a 2-h incubation with anti mouse/rabbit secondary antibodies (CST, Danvers, MA, USA, 7074/7076, 1:1000), and finally, it was exposed using enhanced chemiluminescence (ECL) reagents (Abcam, Cambridge, UK, Ab133406) by a fully automated chemiluminescence imager, Tanon 5200 (Tanon, Shanghai, China). Each WB assay was repeated three times. Details of the antibodies used are provided in Table S2.

### 2.5 Real-Time Quantitative Polymerase Chain Reaction (qPCR)

TRIzol was used to perform the RNA extraction in Penl1, Penl2, 149RM, LM156, and 149RCA, which is followed by reverse transcription with the HiScript QRT SuperMix Kit and amplification with the ChamQ SYBR qPCR Green Master Mix Kit, all in accordance with the manufacturer’s instructions (Vazyme, Nanjing, China, R423-01 and Q411-02). The relative expression of the aimed genes was calculated by the 2^−ΔΔCt^ method and normalized against GAPDH expression. Refer to Table S3 for the list of primers used.

### 2.6 Proliferation Assays

To assess the proliferation of 149RM and Penl2 cells, 2000 PSCC cells (shTROP-2 and NC; TROP-2 OE and vector) were plated in 96-well plates and incubated with Cell Counting Kit-8 (CCK-8, Dojindo, Kumamoto, Japan, CK04) for two hours. Cell counts were calculated with a microplate reader at 450 nm optical density over 7 days with an equation: cell proliferation = (average OD value of experiment day − average OD value of blank hole)/(average OD value of day 1 − average OD value of blank hole). Also, we evaluate the IC_50_ value with the CCK-8 assay. It was calculated with the same equation: cell viability = (average OD value after adding drugs for 72 h − average OD value of blank hole)/(average OD value without drugs − average OD value of blank hole), and IC_50_ represents the drug concentrations when the cell viability is 50%. To perform the colony formation experiment, 3000 cells were seeded in 6-well plates and incubated for half a month, and colonies were dyed with 1% crystal violet at room temperature for 2 h, after which colonies were quantified using ImageJ 2.0 (National Institutes of Health, Bethesda, MD, USA).

### 2.7 Cell Cycle Assay

Cells from the shTROP-2 and shNC groups were cultured in DMEM without serum and fixed overnight in 70% ethanol. Subsequently, PSCC cells were stained using a staining Kit for cell cycle (KeyGEN, Nanjing, China, KGA512). To assess the proportion of PSCC cells in each phase of the cell cycle, flow cytometry (ACEA NovoCyte™, San Diego, CA, USA, NovoCyte Penteon) was conducted, followed by data analysis performed using NovoExpress™ 1.3.1 (Walnut Creek, CA, USA).

### 2.8 Tumorigenesis Model in Nude MICE

The SYSUCC Animal Ethics Committee approved the animal experiments (L102022023001Y). Male BALB/c nude mice, aged six to eight weeks, were obtained from Jiangsu GemPharmatech Co., Ltd. (Nanjing, China). For the tumorigenesis model, 10 nude mice were divided into two groups, and each group contained 5 mice. And 1 million Penl2 cells suspended in 150 µL of saline were injected by 1 mL syringe under the dorsal skin of mice. A single observer monitored the mice’s tumor volume and weight daily. Mice were euthanized if they experienced over 20% weight loss or if tumor size exceeded 2500 mm^3^. Tumors were excised and weighed one week post-formation. The volume of the tumor was calculated by the following equation: volume (mm^3^) = 0.5 ∗ width ∗ width ∗ length.

### 2.9 Toxicity and Targeting Test

To validate the targeting ability of the TROP-2 ADC drug (IMMU-132, MCE, HY-132254). TROP-2 protein was fixed on an ELISA plate (BeyoGold™, Beyotime, FST015), and we diluted the ADC drug into different dilutions. Then we added it into the ELISA plate and detected OD 450 after incubating for 30 min. Also, we labeled the TROP-2 ADC drug with 2 mg/mL Cy-5 fluorescence. After adding labeled Trop-2 ADC drug into TROP-2 OE Penl2 cells for 0 h/2 h/4 h, immunofluorescence (IF) was performed to see the location of ADC drugs with a whole slide scanner (KF-PRO-400-HI, KFBIO, Ningbo, China).

As for the toxicity test. Three groups of nude mice received injections of 200 μL containing 1.75 mg/mL IMMU-132, 10 ng/mL cisplatin (MCE, HY-17394), and pH 7.3 1× PBS. After 3 weeks, the major organs and blood of these nude mice were obtained. The organs were fixed in 4% PFA for 24 h (Beyotime, P0099) and dehydrated with an alcohol gradient of 100%, 90%, 80%, 70%. The organs were embedded in paraffin at 60 degrees for 2 h. The dewaxing and hydration procedure was described in the IHC assay. Then, it was stained with an H&E staining kit (Beyotime, C0105) and scanned with a whole slide scanner (KF-PRO-400-HI, KFBIO, Ningbo, China).

### 2.10 Bioinformatics Analysis

To delineate transcriptomic alterations induced by TROP-2 knockdown, RNA sequencing (RNA-seq) was conducted comparing Penl2 shNC control cells and TROP-2-silenced cells (sh1 line). After extracting RNA from shNC and shTROP-2 cells, significantly differentially expressed genes were subjected to functional annotation using the R-based clusterProfiler package for Gene Ontology (GO) and Kyoto Encyclopedia of Genes and Genomes (KEGG) enrichment analyses.

### 2.11 Statistical Analysis

SPSS software (version 25.0, IBM Corp., Armonk, NY, USA) was applied in this study. Data are presented in the form of means ± SD. Student’s *t*-test or one-way ANOVA was conducted to analyze the differences between the two groups. Chi-square tests were conducted to analyze the association of the two variables. As for survival analysis, Kaplan–Meier survival curves were utilized. Pearson’s chi-square test was used to analyze the associations. A *p-*value of no more than 0.05 was deemed significant.

## 3 Results

### 3.1 TROP-2 Expression in PSCC Tissues and PSCC Cell Lines

**Figure 1:**
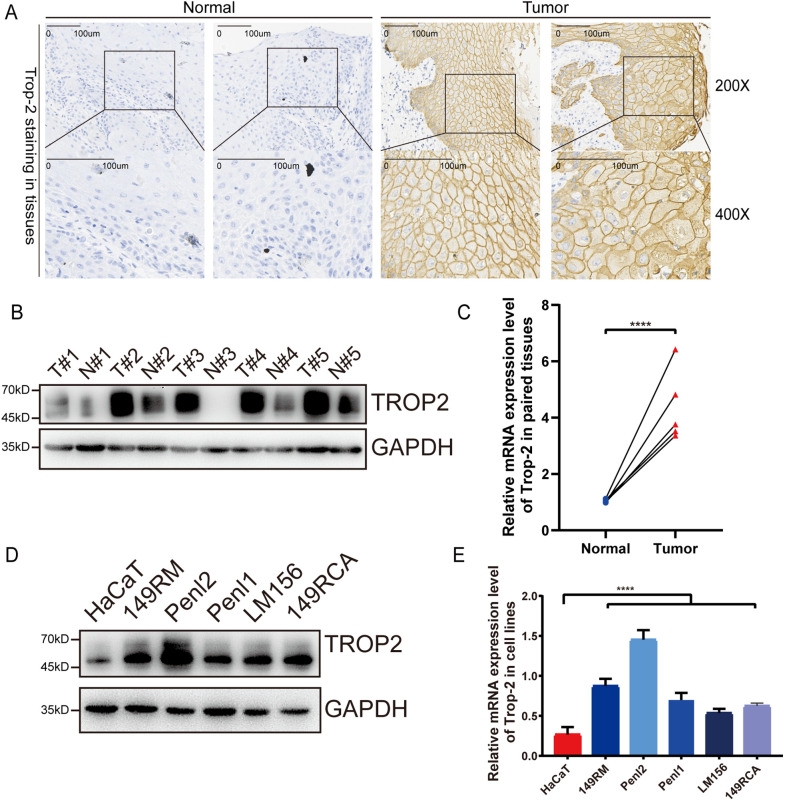

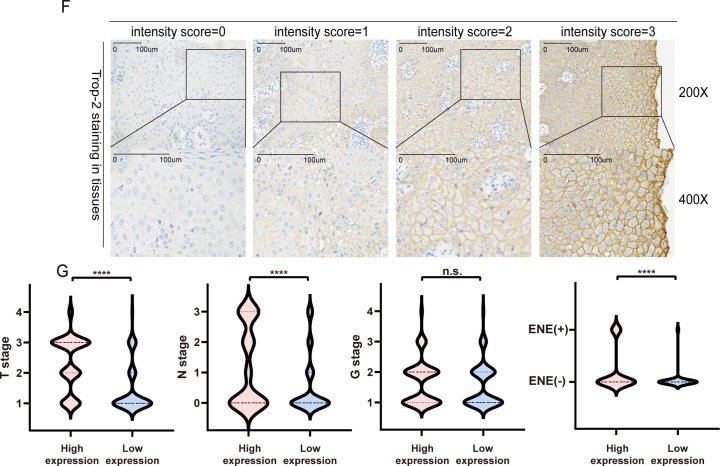
The expression of TROP-2 in PSCC tissues and PSCC cells. (**A**) TROP-2 staining in normal tissue and tumor tissue. (**B,C**) TROP-2 protein and mRNA were higher in tumor tissues compared to normal tissues. (**D,E**) TROP-2 expression in different PSCC cell lines. (**F**) The standard staining intensity score of TROP-2 in tumor fissue in the member was no staining was scored with 0, weak staining was scored with 1, moderate staining was scored with 2, and strong staining was scored with 3. (**G**) Proportion of TROP-2 expression in different subgroups. ****p < 0.0001, n.s., No significant. Statistics are expressed as the means ± SDs of three independent experiments. ENE: Extranodal extension; PSCC: Penile squamous cell carcinoma

IHC was conducted to assess the expression of TROP-2 in paired PSCC and normal tissues. Fig. 1A illustrates that compared to normal tissues, TROP-2 expression is significantly higher in PSCC tissues. Both WB and qPCR assays confirmed elevated TROP-2 expression in tumor tissues (Fig. 1B,C). Additionally, we detected its expression in five PSCC cancer cell lines and normal squamous epithelium, and the results showed the same trend (Fig. 1D,E). These findings collectively demonstrate that TROP-2 is significantly expressed in PSCC tissues and cell lines.

### 3.2 Correlation between TROP-2 Expression and Clinicopathological Characteristics

**Figure 2:**
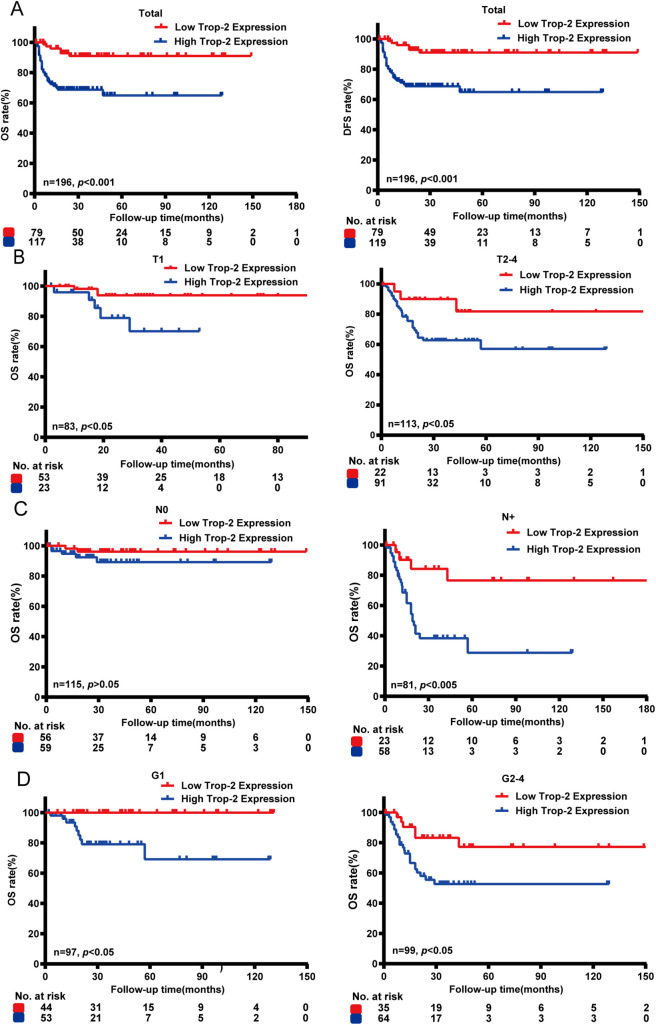
Survival analysis between TROP-2 expression and clinicalopathological factors in PSCC patients. (**A**) Kaplan-Meier survival analysis indicated that high expression of TROP-2 was associated with reduced OS and DFS. (**B**) Survival analyses were also performed in the pT subgroup, (**C**) pN subgroup, and (**D**) the G subgroup. DFS: 5-year disease-free survival; OS: Overall survival

IHC was also conducted on 198 clinical PSCC specimens to confirm TROP-2 expression in cancer tissues. Detailed IHC scoring criteria are provided in the Section 2. As shown in Fig. 1F, TROP-2 was mainly expressed in the cytoplasmic membrane. A total of 60.2% (119/198) of the cohort had high TROP-2 expression, while 39.8% (79/198) had low TROP-2 expression. Chi-square tests indicated that elevated TROP-2 expression correlated with adverse clinical characteristics, such as pT stage, pN stage, clinical stage, and extranodal extension (ENE) (*p* < 0.05) (Fig. 1G and Table S1). The result of Kaplan-Meier survival analysis revealed that elevated expression of TROP-2 correlates with reduced overall survival (OS) and 5-year disease-free survival (DFS) in PSCC patients (*p* < 0.05) (Fig. 2A).

To explore the association between TROP-2 expression and the outcome of patients at different clinical stages, we divided PSCC patients into different subgroups. Subgroup analysis indicated that elevated TROP-2 expression is related to poor outcome in both ≤pT1 and pT2–4 stage subgroups (*p* < 0.05). In the G stage subgroup, high expression of TROP-2 is related to poor outcome in both G1 and G2–4 patients (*p* < 0.05). In the N stage subgroup, high TROP-2 expression correlated with poor outcomes exclusively in pN+ stage patients (*p* < 0.05), while no such association was observed in pN0 patients (*p* > 0.05). This suggests a stronger link between TROP-2 expression and prognosis in cases of lymph node metastasis (Fig. 2). With the above findings, it is highly possible that TROP-2 provides additional prognostic value in pN+ PSCC patients rather than in pN0 patients.

### 3.3 TROP-2 Promotes PSCC Cell Proliferation In Vitro

**Figure 3:**
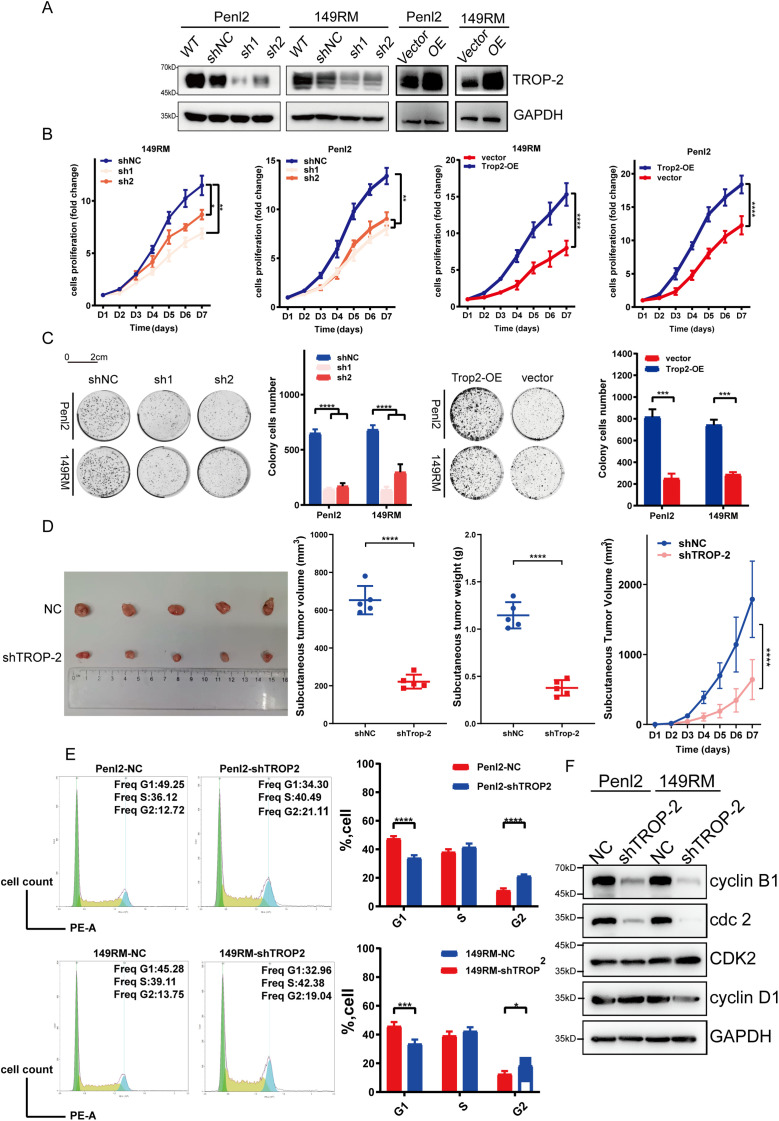
TROP-2 promotes cell proliferation in PSCC *in vitro* and *in vivo*. After being transfected with silencing or over-expressing plasmids, the following experiments were conducted to determine whether TROP-2 affects cell proliferation. (**A**) WB for TROP-2 expression in TROP-2 knockdown/over-express PSCC cells. (**B**) Knockdown and overexpression of TROP-2 in PSCC cells regulate cell proliferation in the CCK-8 experiment *in vitro*. (**C**) Knockdown and overexpressed of TROP-2 in PSCC cells regulate cell proliferation in the cell colony formation experiment (**D**) and regulate the growth of the tumor *in vivo*. (**E**) PSCC cells with TROP-2 knockdown exhibit an increased percentage in the G2 phase. (**F**) The knockdown of TROP-2 decreased the G2/M checkpoint proteins expression, such as cdc2 and cyclin B, while G1/S regulators remained unchanged. Significance levels are indicated as follows: *p < 0.05; **p < 0.01; ***p < 0.001; ****p < 0.0001. Statistics are presented as the means ± SDs of three independent experiments. NC: Negative control; OE: Overexpression; WT: Wild type

To explore the function of TROP-2 in PSCC cells, TROP-2 knockdown and overexpression cell lines were created in 149RM and Penl2 cells using plasmid technology (Fig. 3A). CCK-8 kit assays identified that knockdown of TROP-2 inhibited growth, while its over-expression strongly promoted the growth of PSCC cells (Fig. 3B). A cell colony formation assay demonstrated that TROP-2 knockdown resulted in smaller and fewer colonies compared to the control group, whereas TROP-2 overexpression produced the opposite outcome (Fig. 3C). Overall, the findings suggest that TROP-2 enhances the growth and proliferation of PSCC cells.

### 3.4 TROP-2 Promotes PSCC Cell Proliferation In Vitro

To explore whether TROP-2 has similar proliferative effects *in vivo*, animal tumor models were established by injecting nude mice with PSCC cells percutaneously. Fig. 3D illustrates that tumors in the TROP-2 group were significantly lighter and smaller than the control group, suggesting that TROP-2 enhances PSCC growth *in vivo*.

### 3.5 TROP-2 Enhances the Cell Proliferation of PSCC by Regulating the G2/M Phase

To investigate the mechanism by which TROP-2 promotes the cell proliferation of PSCC, the role of TROP-2 in cell cycle regulation was examined. Knockdown of TROP-2 elevated the percentage of 149RM and Penl2 in G2 phase, as shown in Fig. 3E. The WB results indicated that in TROP-2-overexpressing PSCC cells, G2/M phase regulators cyclin B1 and cdc2 were downregulated, whereas G1/S phase regulators CDK2 and cyclin D1 remained unchanged (Fig. 3F). This suggests that TROP-2 knockdown inhibits PSCC cell growth by arresting the cell cycle at the G2/M phase.

### 3.6 TROP-2 Modulates the G2/M Cell Cycle Transition through the AKT Signaling Pathway

**Figure 4:**
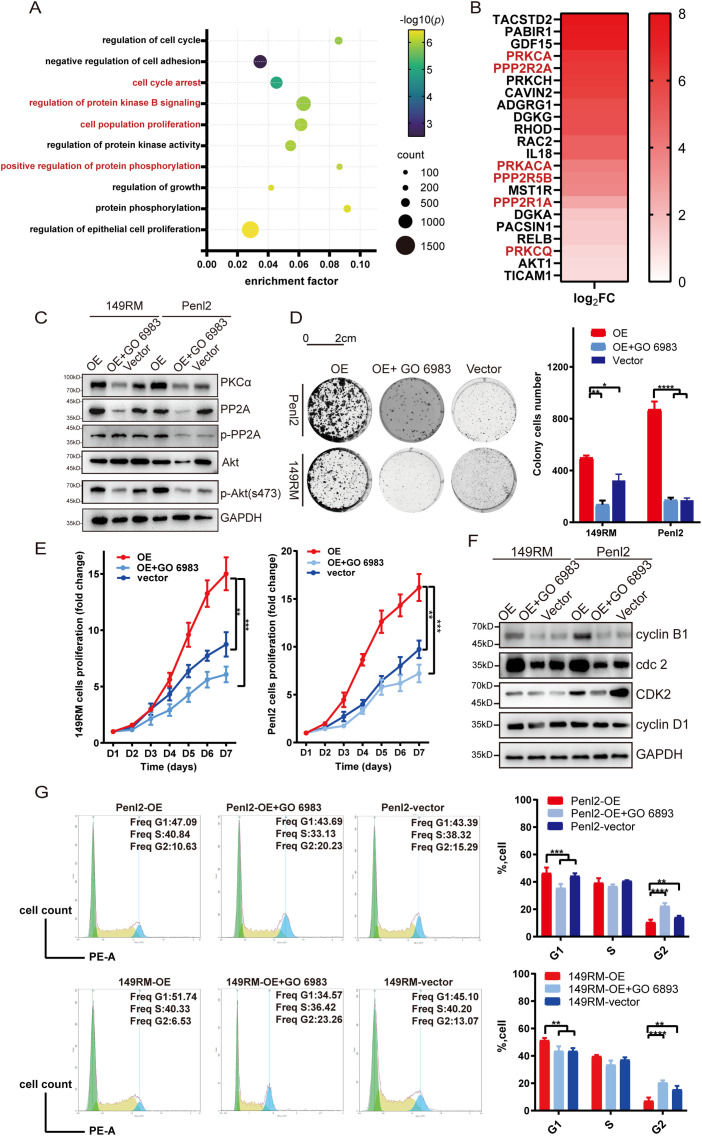
TROP-2 up-regulated PSCC proliferation through PKCa and AKT pathway. (**A,B**) The GO analysis and heatmap of transcriptome sequencing. (**C**) The expression of protein in the PKCa and AKT pathways in PSCC cells after using PKCa inhibitors. (**D,E**) Cell proliferation was rescued in TROP-2 over-expressed PSCC cells after using PKCa inhibitors. (**F,G**) PSCC cells were arrested in G2/M stage after using PKCa inhibitors. *p < 0.05; **p < 0.01; ***p < 0.001; ****p < 0.0001. Statistics are presented as the means ± SDs of three independent experiments. FC: Fold change; OE: Overexpression

To investigate the mechanism by which TROP-2 enhances PSCC cell growth, we performed transcriptome sequencing in shTROP-2 Penl2 and control cells. Gene Ontology (GO) enrichment analyses indicated significant enrichment in cell cycle regulation, cell proliferation, AKT signaling, protein phosphorylation, and protein kinase activity upon TROP-2 inhibition in PSCC (Fig. 4A). According to the heatmap, knockdown of TROP-2 in Penl2 cells down-regulated several proteins in the PKCα and AKT pathways, such as PKCα, PP2A, p-PP2A and AKT (Fig. 4B). TROP-2 may promote AKT hyperphosphorylation at S473 by activating protein kinase Cα (PKCα), reducing Ca^2+^-dependent PP2A phosphatase activity, and decreasing AKT dephosphorylation, thereby inducing cell proliferation [18,19]. Therefore, we investigated the expression of PKCα, PP2A, p-PP2A, and AKT in this pathway. Our findings proved that the TROP-2 overexpression causes upregulation of PKCα, p-PP2A, and AKT compared to the vector group (Fig. 4C). To further explain that TROP-2-mediated cell cycle progression relies on the PKCα and AKT pathways, we added a PKCα inhibitor (GO 6983, MCE) to TROP-2-overexpressed PSCC cells. As shown by the WB results, as the PKCα pathway was inhibited, the up-regulation of AKT-p473 in TROP-2-overexpressed group turned into down-regulation (Fig. 4C). Similarly, when GO 6983 was added to TROP-2-overexpressed PSCC cells, the increase in cell proliferation also decreased (Fig. 4D,E). These findings revealed that TROP-2 regulated cell growth in PSCC cells by decreasing the dephosphorylation of AKT. Our cell cycle rescue experiment demonstrated that inhibiting PKCα causes an obvious increase in the percentage in G2 phase, along with decreased expression of cdc2 and cyclin B1. This result indicates that PSCC cells are arrested in the G2/M phase upon AKT inhibition (Fig. 4F,G).

### 3.7 The TROP-2 ADC Demonstrates Notable Efficacy in PSCC Cells In Vivo and In Vitro

**Figure 5:**
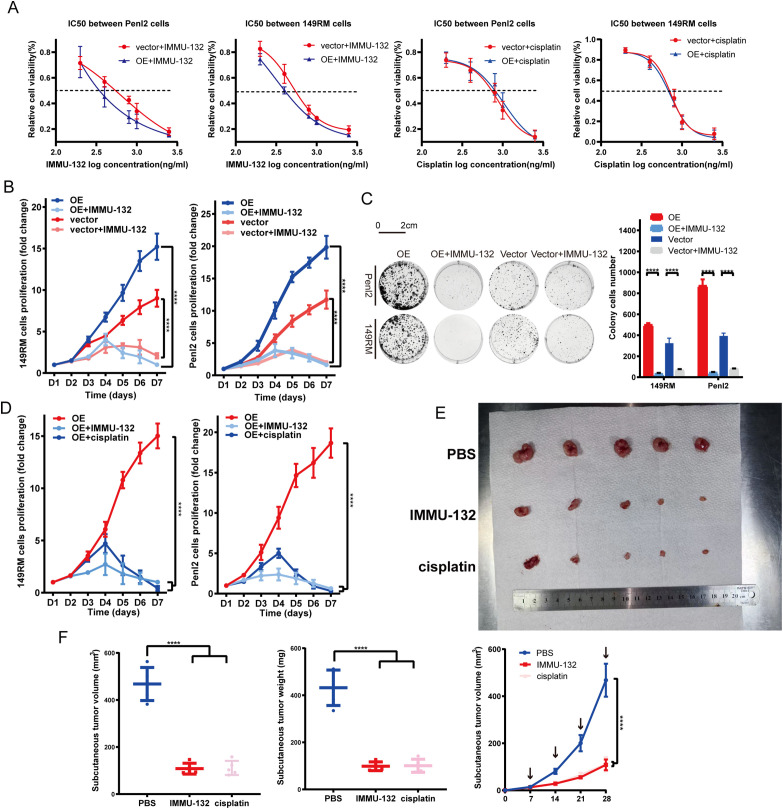
TROP-2 ADC drug shows a significant efficacy in PSCC cells *in vivo* and *in vitro*. (**A**) TROP-2 over-expressed PSCC cells showed a lower IC_50_ dose to ADC drug than the negative control group. (**B,C**) TROP-2 ADC drug down-regulated the cell proliferation of PSCC cells more significantly in the TROP-2 over-expressed group. (**D–F**) TROP-2 ADC drug also showed better efficacy than cisplatin in TROP-2 over-expressed cells *in vivo*. ****p < 0.0001. Statistics are presented as the means ± SDs of three independent experiments. IC50: Half maximal inhibitory concentration; OE: Overexpression

Given the association of TROP-2 to adverse outcomes and heightened proliferation in PSCC, we performed drug experiments to evaluate the efficacy of the TROP-2 ADC (IMMU-132, MCE) *in vivo* and *in vitro*. As shown by the IC_50_ test, Penl2 and 149RM TROP-2 over-expressed cells had lower IC-50 values, indicating greater sensitivity to IMMU-132 (Fig. 5A). Colony formation and CCK-8 assays demonstrated that IMMU-132 significantly inhibited cell proliferation in overexpressed cells compared to negative control cells (Fig. 5B,C). We evaluated the potential of IMMU-132 as an alternative to traditional chemotherapy agents like cisplatin by comparing their efficacy *in vitro* and *in vivo*. Results indicated that IMMU-132 demonstrated comparable efficacy to cisplatin in both settings (Fig. 5D–F).

### 3.8 The Toxicity and Targeting Test of IMMU-132

**Figure 6:**
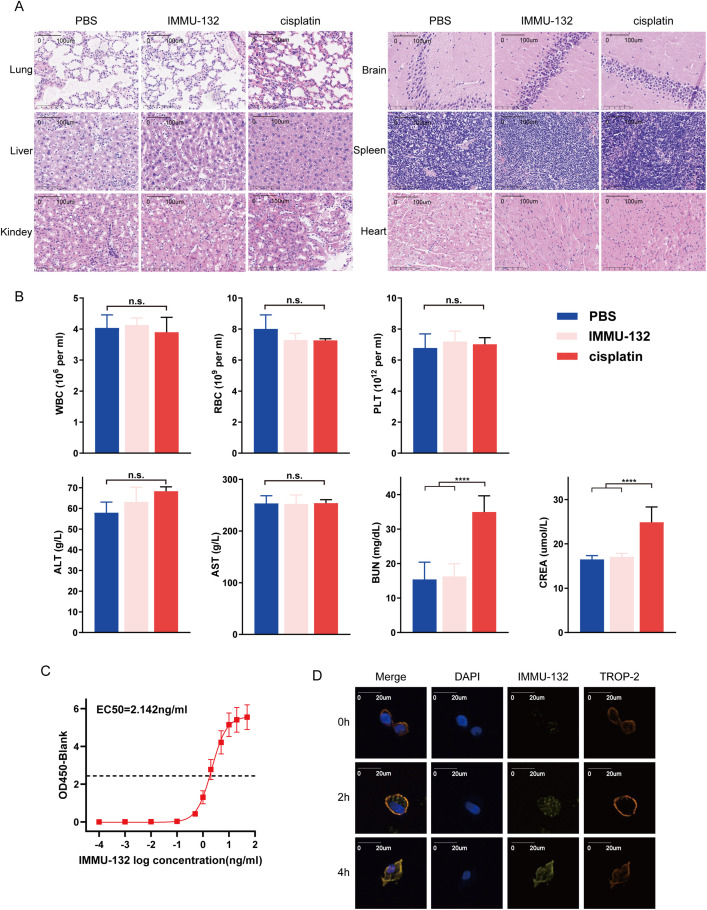
The toxicity and targeting test of TROP-2 ADC drugs (IMMU-132). IMMU-132 didn’t show serious toxicity to major organs of mice, and its targeting function was also verified. (**A,B**) After injecting PBS, cisplatin or IMMU-132 for 3 weeks, the major organs and blood of mice were obtained. Results of H&E staining and biochemical examinations showed that IMMU-132 didn’t cause obvious toxicity to mice compared with PBS, while cisplatin caused kidney toxicity. (**C**) TROP-2 protein was fixed on the ELISA plate, and different concentrations of IMMU-132 were used to examine its binding ability to TROP-2 protein. (**D**) IMMU-132 was labeled with CY-5 fluorescence. Immunofluorescence was performed at different time points after IMMU-132 was used in TROP-2 OE Penl2 cells. ****p < 0.0001, n.s., No significant. Statistics are expressed as the means ± SDs of three independent experiments

To evaluate IMMU-132 toxicity, we treated healthy nude mice with PBS, cisplatin or IMMU-132 for three weeks and obtained their major organs (Fig. S2). H&E staining of the kidney, liver, heart, brain, spleen, and lung showed no significant pathological changes or inflammatory infiltration in the IMMU-132 group compared to the PBS group, while mice kidney in cisplatin group showed renal tubular injury and vacuolar degeneration in renal cell with varying degrees (Fig. 6A). Additionally, biochemical examinations were conducted, which indicated that mice in cisplatin group suffered from renal injury compared with mice in IMMU-132 group and PBS group (Fig. 6B). These findings indicate that IMMU-132 has mild side effects and cause less damage to major organs, especially kidney, but has efficacy similar to that of cisplatin.

To verify whether IMMU-132 can bind to TROP-2 protein specifically. ELISA and IF were performed. As is shown in Fig. 6C, there is a positive correlation between dilution of IMMU-132 and OD 450, with an EC50 of 2.142 ng/mL. Also, according to Fig. 6D, IMMU-132 can be enriched in TROP-2 OE Penl2 cells as time went by, which indicated that IMMU-132 can target TROP-2 expressing cells specifically.

## 4 Discussion

According to European Association of Urology (EAU) guidelines, advanced PSCC is a severe disease with poor outcome, for which surgery combined with adjuvant chemotherapy is recommended [1]. However, the first-line chemotherapy TIP regimen only has a moderate OR of 40% and might cause severe side effects that some patients might fail to tolerate [6,20]. Once an intolerant side effect occurs, the available second-line therapy, paclitaxel monotherapy, only has a response rate of 30% and no survival, which makes new exploration for second-line therapy urgently needed [21].

ADC drugs are novel treatments that consist of cytotoxic drugs and conjugated tumor-targeting antibodies and are able to transport a cytotoxic payload to target cells precisely [22–24]. According to several clinical studies, ADC drugs have been proven to achieve satisfactory therapeutic effects in various tumors with minimal side effects [25,26]. The use of ADC drugs in penile cancer is constrained by the absence of reliable targets. Therefore, exploring potential targets of ADC drugs in advanced PSCC patients is instrumental for identifying treatment options for advanced PSCC patients.

TROP-2 is a transducer of intracellular calcium signals that contributes to proliferation, transformation, and self-renewal in many human cancers and is upregulated in various solid cancers, especially epithelial carcinomas [27–29]. This research found that compared to normal tissues, expression of TROP-2 was notably higher in PSCC tissues, with 60.2% of patients showing elevated TROP-2 levels. The statistical analysis indicates that elevated TROP-2 expression correlates with poorer overall survival and adverse clinical and pathological features, including pT and pN status, clinical stage, and ENE, suggesting TROP-2 as a potential prognostic biomarker for PSCC patients.

To further understand whether TROP-2 expression is a potential prognostic factor in patients of different clinical statuses. Subgroup analysis indicated that TROP-2 expression is associated with poor outcome in both the T stage and G stage subgroups. In the pN0 subgroup of the N stage, OS did not significantly differ between groups with low and high TROP-2 expression. pN0 patients may achieve clinical cure through local excision, often resulting in a favorable outcome, and their 5-year OS is up to 90% [30], which could be a significant factor. In contrast, TROP-2 was strongly associated with poor outcomes in the pN+ subgroup. Numerous studies indicate that OS significantly declines in pN+ patients due to lymph node metastasis (LNM), a critical prognostic factor in PSCC, with survival rates of N0 at 85%–100%, N1 at 79%–89%, N2 at 17%–60%, and N3 at 0%–17% [3,31]. However, there are arguments about the current N stage, and according to Oliver et al., long-term survival does not differ significantly between N1 and N2 disease. Our study indicates that high TROP-2 expression is linked to LNM characteristics, including advanced pN stage and ENE, and is associated with prognosis in the pN+ subgroup, suggesting its prognostic value in pN+ patients but not in pN0 patients.

In addition, we investigated the role of TROP-2 in the biological function of PSCC cells. The findings indicated that TROP-2 knockdown markedly suppressed PSCC cell proliferation by inducing cell cycle arrest at the G2/M phase. Then, transcriptome sequencing was performed, and the PKCα- and AKT-mediated signalling pathways were significantly enriched according to the GO analysis, as was cell growth regulation. This result revealed that TROP-2 upregulates proliferation by regulating the AKT-mediated signalling pathway.

The AKT signaling pathway, a serine/threonine kinase, is crucial in the PI3K pathway, where its phosphorylation and activation prevent cell cycle arrest and enhance cell proliferation [19,32,33]. Guerra et al. also noted that TROP-2 promoted the growth of cancer through the AKT pathway [18]. It was hypothesized that TROP-2 overexpression elevates intracellular Ca^2+^, activating PKCα, which phosphorylates PP2A at Ser41, reducing its activity and subsequently decreasing AKT dephosphorylation [34,35]. Correspondingly, this study revealed that the expression of PKCα, p-PP2A, and p-AKT increased as TROP-2 was overexpressed. Application of GO 6983 to TROP-2-overexpressing cells restored levels of p-PP2A and p-AKT in this group. When Go 6983 was applied, the cell cycle and cell proliferation in the overexpression group were also rescued. Therefore, TROP-2 upregulates the proliferation of PSCC cells by preventing the dephosphorylation of AKT.

The use of ADC drugs in penile cancer is constrained by the absence of reliable targets. Given that TROP-2 is overexpressed in 60.2% of PSCC patients and enhances PSCC cell proliferation, it might be a suitable target for ADC therapy in advanced PSCC cases. In fact, TROP-2 has become a hot research topic as a target for ADC treatment. IMMU-132, an ADC targeting TROP-2, demonstrates anticancer effects on various cancers, including bladder, pancreatic, and gastric cancers [15,36]. To assess the efficacy of TROP-2 ADC in PSCC cells, we conducted various functional experiments. IC_50_ tests revealed that TROP-2-overexpressed cells had a lower IC_50_ than those in the vector group, indicating that the ADC drugs tended to have better anticancer effects on TROP-2-overexpressed cells. The study investigated the therapeutic potential of IMMU-132 relative to the TIP regimen by comparing their efficacy with cisplatin. The findings indicated that TROP-2 ADC drugs exhibited comparable cytotoxic effects on PSCC cells to cisplatin in both *in vivo* and *in vitro* settings. More importantly, no major organ toxicity or side effects were observed in nude mice treated with IMMU-132, while some studies revealed that injecting cisplatin might cause renal inflammation, which might lead to chronic kidney injury characterized by renal fibrosis [37–39]. These findings support that the TROP-2 ADC could be a competent alternative for these patients who could not bear the traditional TIP regimen or fail to achieve a steady response to TIP treatment.

There also exist several limitations in our study. First, this study didn’t include a large sample of patients because PSCC is a rare cancer, but we will continually include more patients in the future to further explore the role of TROP-2 in PSCC. Secondly, the complete TIP regimen isn’t performed in mice model because mice can’t tolerate the toxicity of the whole TIP regimen, making it less convincing.

This study concludes that elevated TROP-2 expression correlates with adverse clinicopathological features and negative outcomes. It can promote the growth of PSCC cells via the AKT pathway. The TROP-2 ADC demonstrates comparable efficacy to cisplatin in both *in vivo* and *in vivo* assays, indicating the potential of TROP-2 as a novel target for advanced PSCC patients.

## Supplementary Materials

Figure S1 Cut-off value determined by X-tile. (A-B) X-tile determined the cutoff value for the TROP-2 expression. (C) Following survival analysis improved the cut-off value divided PSCC patients into two groups with different survival.

Figure S2 Major organs of mice after injecting PBS, cisplatin or IMMU-132.

## Data Availability

The datasets supporting the conclusions of this article are available from the corresponding authors on reasonable request. The authenticity of this article has been validated by uploading the key raw data onto the Research Data Deposit platform (www.researchdata.org.cn).

## Supplementary Materials

The supplementary material is available online at (https://www.techscience.com/doi/10.32604/or.2025.066184/s1).

## List of Abbreviations

ADCs: Antibody drug conjugates
CCK-8: Cell Counting Kit-8
DMEM: Dulbecco’s modified Eagle’s medium
DFS: Five-year disease-free survival
ECL: Enhanced chemiluminescence
ENE: Extranodal extension
HRP: Horseradish peroxidase
IC_50_: Half maximal inhibitory concentration
IHC: Immunohistochemistry
LNM: Lymph node metastasis
NC: Negative control
OE: Over expression
OR: Objective response
OS: Overall survival
PLNM: Pelvis lymph node metastasis
PSCC: Penile squamous cell carcinoma
PKCα: Protein kinase Cα
qPCR: Quantitative real-time polymerase chain reaction
SG: Sacituzumab govitecan
shRNA: Short hairpin RNA
WB: Western blot
WT: Wild type
