# Supplementary figures and images for: TROP-2 Promotes Cell Proliferation via the AKT-Mediated PKCα Pathway and Is a Novel Target for Antibody-Drug Conjugates in Penile Carcinoma
Source: Oncol Res. 2025 Nov 27;33(12):3973–89. doi: 10.32604/or.2025.066184 (PMC12712658; doi:10.32604/or.2025.066184)

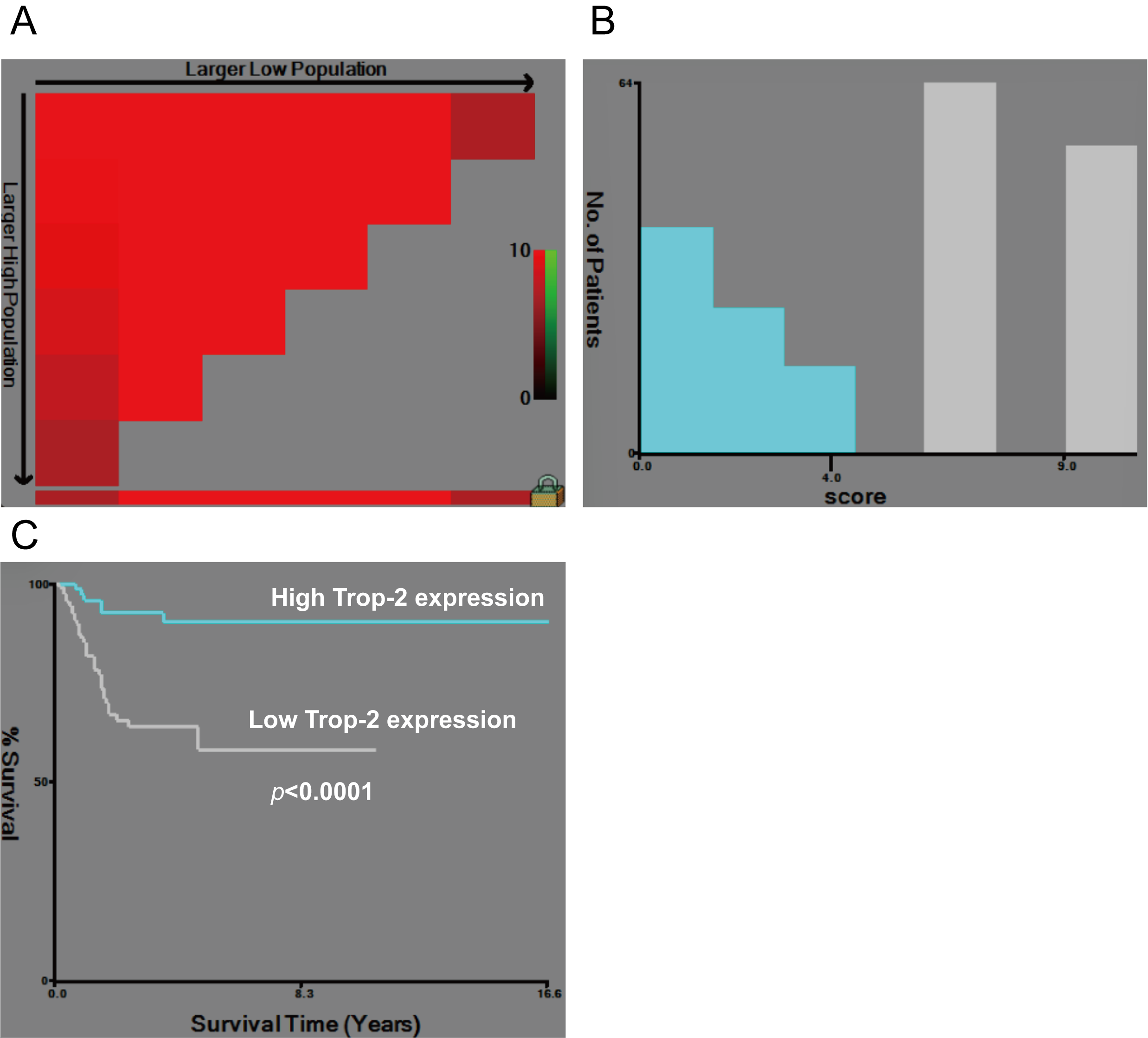

Supplement: Figure S1 [file OncolRes-33-66184-s001.tif]

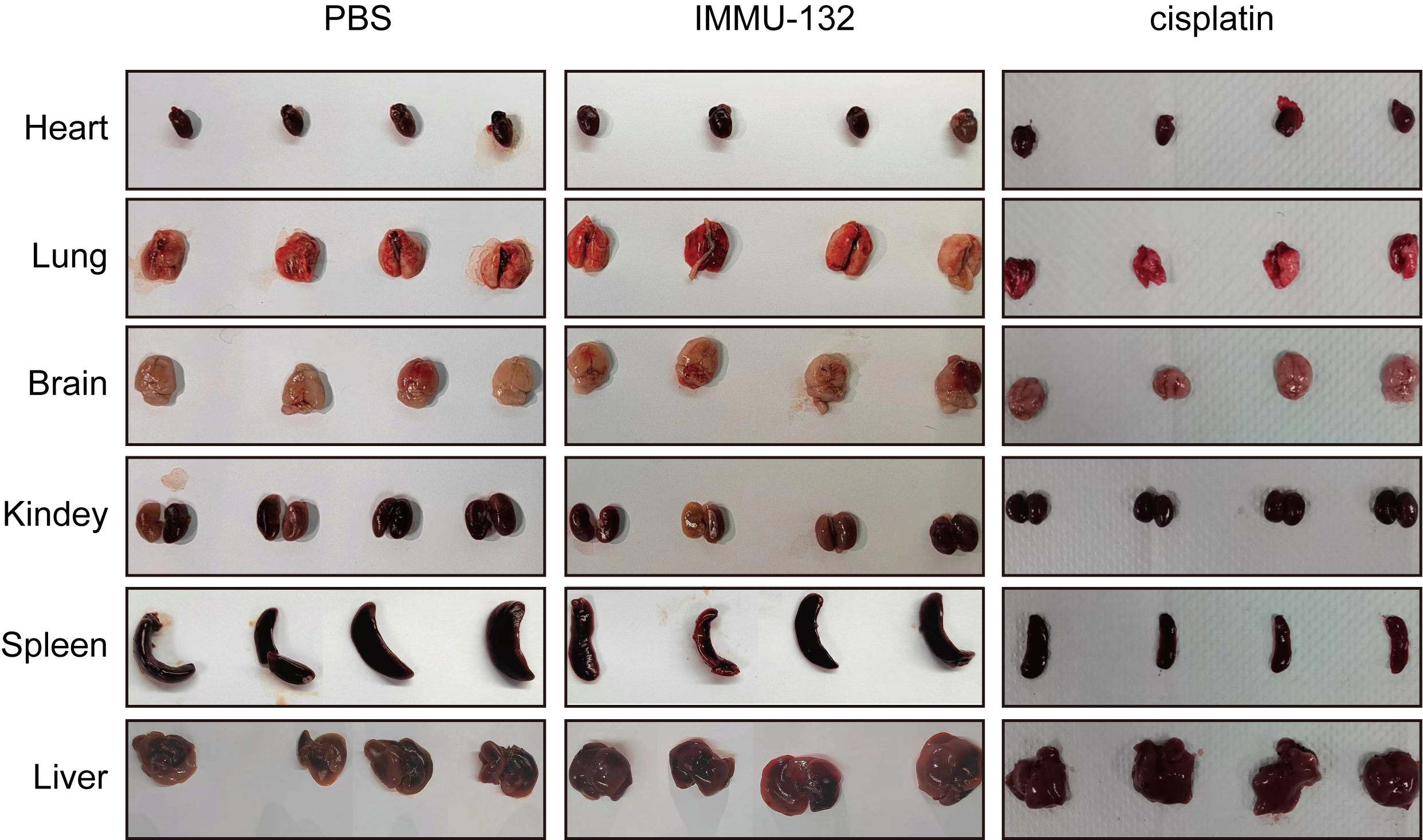

Supplement: Figure S2 [file OncolRes-33-66184-s002.tif]
