# Supplementary material for: TROP-2 Promotes Cell Proliferation via the AKT-Mediated PKCα Pathway and Is a Novel Target for Antibody-Drug Conjugates in Penile Carcinoma
Source: Oncol Res. 2025 Nov 27;33(12):3973–89. doi: 10.32604/or.2025.066184 (PMC12712658; doi:10.32604/or.2025.066184)
Supplement: Supplementary file 3 [file OncolRes-33-66184-s003.docx]

*Table S1* Chi-square tests revealed that high TROP-2 expression was associated with poor clinical features, including pT stage, pN stage, clinical stage and ENE.

|  |  | TROP-2 IHC staining | |  |  |
| --- | --- | --- | --- | --- | --- |
|  | PSCC cohort  (N=196) | Low expression  (N=83), 41.9% | High expression  (N=115), 58.1% | χ^2^ | *p*-value |
| Age |  |  |  | 0.010 | 0.992 |
| <55 | 92 (46.9) | 37(18.8) | 55 (28.0) |  |  |
| ≥55 | 104 (53.0) | 42 (21.4) | 62 (31.6) |  |  |
| pT status |  |  |  | 42.984 | < 0.001 |
| ≤pT1 | 76 (38.7) | 53 (27.0) | 23(11.7) |  |  |
| pT2 | 39 (19.9) | 10 (5.1) | 29 (14.7) |  |  |
| pT3 | 68 (34.6) | 11 (5.6) | 57 (29.1) |  |  |
| pT4 | 6 (3.0) | 1 (0.5) | 5 (2.5) |  |  |
| Tx | 7 (3.6) | 4 (2.0) | 3 (1.5) |  |  |
| pN status |  |  |  | 11.410 | < 0.05 |
| N0 | 115 (58.6) | 56 (28.5) | 59 (30.1) |  |  |
| N1 | 19 (9.7) | 10 (5.1) | 9 (4.6) |  |  |
| N2 | 24 (12.2) | 6 (3.0) | 18 (9.2) |  |  |
| N3 | 38 (19.4) | 7 (3.6) | 31 (15.8) |  |  |
| Metastasis |  |  |  | 0.092 | 0.761 |
| M0 | 193 (98.4) | 79 (40.3) | 114 (58.1) |  |  |
| M1 | 3 (1.5) | 0 (0) | 3 (1.5) |  |  |
| Clinical stage |  |  |  | 31.937 | < 0.001 |
| Stage Ⅰ | 46 (23.4) | 35 (17.8) | 11 (5.6) |  |  |
| Stage Ⅱ | 68 (34.7) | 21 (10.7) | 47 (24.0) |  |  |
| Stage Ⅲ | 43 (21.9) | 16 (8.2) | 27 (13.7) |  |  |
| Stage Ⅳ | 38 (19.4) | 7 (3.6) | 31 (15.8) |  |  |
| Histology |  |  |  | 1.023 | 0.600 |
| G1 | 97 (49.4) | 44 (22.4) | 53 (27.0) |  |  |
| G2 | 76 (38.7) | 27 (13.8) | 49 (24.9) |  |  |
| G3 | 23 (11.7) | 8 (4.1) | 15 (7.6) |  |  |
| ENE |  |  |  | 11.346 | < 0.05 |
| No | 171 (87.2) | 76 (38.8) | 95 (48.4) |  |  |
| Yes | 25 (12.8) | 3 (1.5) | 22 (11.3) |  |  |
| PLNM |  |  |  | 1.502 | 0.220 |
| No | 187 (95.4) | 77 (39.3) | 110 (56.1) |  |  |
| Yes | 9 (4.6) | 2 (1.0) | 7 (3.6) |  |  |

*ENE: Extranodal extension; PSCC: Penile squamous cell carcinoma; PLNM: Pelvis lymph node metastasis.

Table S2 Details of used antibodies

| Antibody | Company | Catalogue number | Dilution |
| --- | --- | --- | --- |
| TROP-2 | CST | E8Y8S | 1:1000 |
| GAPDH | CST | D16H11 | 1:5000 |
| Cyclin B1 | CST | D5C10 | 1:1000 |
| cdc2 | CST | POH1 | 1:1000 |
| CDK2 | CST | E8J9T | 1:1000 |
| Cyclin D1 | CST | E3P5S | 1:1000 |
| PKCα | CST | #2056 | 1:1000 |
| PP2A | Abcam | ab32065 | 1:1000 |
| p-PP2A | Thermo Fisher | PA5-36874 | 1:1000 |
| Akt | CST | C67E7 | 1:1000 |
| p-Akt | CST | D9E | 1:1000 |

Table S3 Details of the used primers.

| Gene name | Forward sequence (5' - 3') | Reverse sequence (5' - 3') |
| --- | --- | --- |
| TROP-2 | ACAACGATGGCCTCTACGAC | GTCCAGGTCTGAGTGGTTGAA |
| GAPDH | GGAGCGAGATCCCTCCAAAAT | GGCTGTTGTCATACTTCTCATGG |
| CYCLIN B1 | AATAAGGCGAAGATCAACATGGC | TTTGTTACCAATGTCCCCAAGAG |
| CYCLIN D1 | GCTGCGAAGTGGAAACCATC | CCTCCTTCTGCACACATTTGAA |
| CDC2 | AAACTACAGGTCAAGTGGTAGCC | TCCTGCATAAGCACATCCTGA |
| CDK2 | CCAGGAGTTACTTCTATGCCTGA | TTCATCCAGGGGAGGTACAAC |


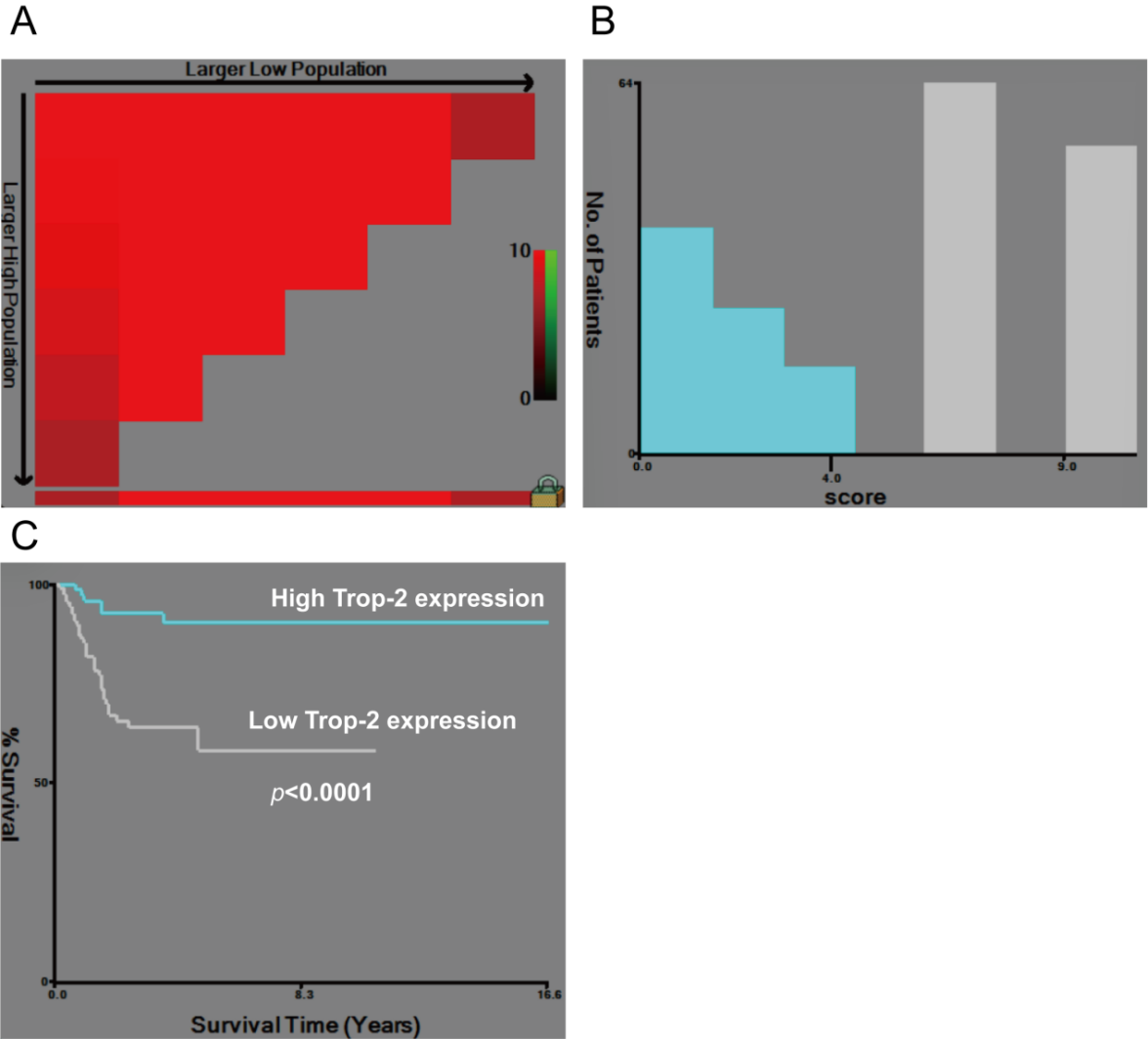


*Fig S1. Cut-off value determined by X-tile.* (A-B) X-tile determined the cutoff value for the TROP-2 expression. (C) Following survival analysis improved the cut-off value divided PSCC patients into two groups with different survival.


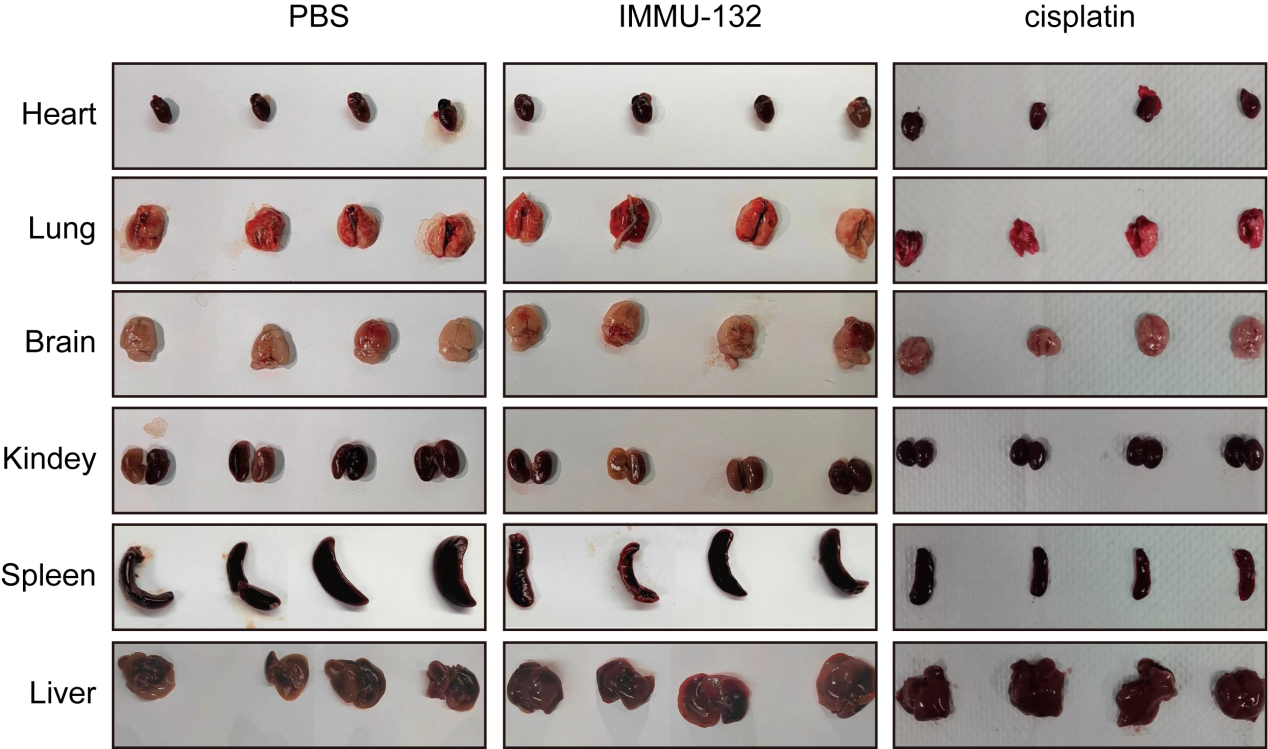


*Fig S2. Major organs of mice after injecting PBS, cisplatin or IMMU-132.*
